# Longitudinal changes in cardiovascular disease–related proteins in welders

**DOI:** 10.1007/s00420-024-02086-8

**Published:** 2024-07-03

**Authors:** Ulrike Maria Dauter, Anda Roxana Gliga, Maria Albin, Karin Broberg

**Affiliations:** 1 Institute of Environmental Medicine, Karolinska Institute, Nobels Väg 13, 171 77 Stockholm, Sweden; 2 Centre for Occupational and Environmental Medicine, Region Stockholm, Sweden; 3 Division of Occupational and Environmental Medicine, Department of Laboratory Medicine, Lund University, Lund, Sweden

**Keywords:** CVD, Welding, Respirable dust, Occupational exposure limit

## Abstract

**Objective:**

Occupational exposure to welding fumes is linked to a higher risk of cardiovascular disease; however, the threshold exposure level is unknown. Here, we aimed to identify changes in proteins associated with cardiovascular disease in relation to exposure to welding fumes.

**Methods:**

Data were obtained from two timepoints six years apart for 338 non-smoking men (171 welders, 167 controls); of these, 174 (78 welders, 96 controls) had measurements available at both timepoints. Exposure was measured as personal respirable dust (adjusted for personal protective equipment), welding years, and cumulative exposure. Proximity extension assays were used to measure a panel of 92 proteins involved in cardiovascular processes in serum samples. Linear mixed models were used for longitudinal analysis. The biological functions and diseases related to the identified proteins were explored using the Ingenuity Pathway Analysis software.

**Results:**

At both timepoints, the median respirable dust exposure was 0.7 mg/m^3^ for the welders. Seven proteins were differentially abundant between the welders and controls and increased incrementally with respirable dust: FGF23, CEACAM8, CD40L, PGF, CXCL1, CD84, and HO1. CD84 was significant after adjusting for multiple comparisons. These proteins have been linked to disorders of blood pressure, damage related to clogged blood vessels, and chronic inflammatory disorders.

**Conclusion:**

Exposure to mild steel welding fumes below current occupational exposure limits for respirable particles and welding fumes in Europe and the US (1–5 mg/m^3^) was associated with changes in the abundance of proteins related to cardiovascular disease. Further research should evaluate the utility of these proteins as prospective biomarkers of occupational cardiovascular disease.

**Supplementary Information:**

The online version contains supplementary material available at 10.1007/s00420-024-02086-8.

## Introduction

Welding generates primary particles of around 5–40 nm in diameter, which form agglomerates of 0.1–1 µm in diameter (Sjögren et al. 2020). Mild steel welding is the major type of welding worldwide (90%), while stainless steel welding is less common (approx. 10%) (Taube 2013). Fumes from mild steel welding consist predominantly of iron (Fe) and manganese (Mn) particles; fumes from stainless steel welding also contain chromium (Cr) and nickel (Ni) (Leonard et al. 2010).

Particles in welding fumes pose a major concern for the occupational exposure of welders to hazardous materials. Indeed, the International Agency for Research on Cancer classified exposure to all types of welding fumes as a Group 1 carcinogen (carcinogenic to humans) in 2017 (International Agency for Research on Cancer (IARC) 2018). Recent studies in welders have also found an increased risk of cardiovascular disease (CVD), such as ischemic heart disease (Mocevic et al. 2015; Sjögren et al. 2020) and myocardial infarction (Mocevic et al. 2015), and an increase of systolic and diastolic blood pressure (Taj et al. 2020). In addition, cross-sectional and longitudinal studies have reported higher blood pressure in welders (Li et al. 2015a; Taj et al. 2020), impairments of cardiac autonomic function (Zhang et al. 2017), changes in heart rate and heartbeat (Cavallari et al. 2016), and damage to coronary artery epithelial cells (Lai et al. 2016). Experiments in mice exposed to stainless steel welding particles revealed systemic inflammation and increased plaque progression (Antonini et al. 2006; Erdely et al. 2011). The Nordic Expert Group for Criteria Documentation of Health Risks from Chemicals has concluded that there is moderately strong evidence of an increased risk of ischemic heart disease associated with exposure to welding fumes (Sjögren et al. 2020).

Although these studies showed a link to CVD, the exact levels that increase risk of CVD remain unknown. The Swedish occupational exposure limit (OEL) for respirable dust is 2.5 mg/m^3^, and many other countries have an OEL at or below 5 mg/m^3^ (GESTIS 2023); however, negative health effects in welders have been observed at average exposures below 1 mg/m^3^. These included an increase of epigenetic markers linked to lung cancer (Dauter et al. 2021), and an increased incidence of chronic obstructive pulmonary disease (Koh et al. 2015). Therefore, setting health-based OELs requires more information on the effects of exposure to welding fumes.

Unique proteins related to cardiovascular processes and diseases can now be measured in small amounts of blood serum or plasma (Assarsson et al. 2014). The proteins have been linked to e.g. inflammation, hypertension, and atherosclerosis, and can serve as biomarkers of effect of exposures (Alhamdow et al. 2019; Gliga et al. 2023).

Here, we measured exposure to welding fumes and explored a selected panel of CVD-related proteins in welders after low-to-moderate exposure to mild steel welding in a southern Swedish cohort.

## Methods

### Study design

In 2010, a cohort of male non-smoking welders and controls was established in southern Sweden (Södra sjukvårdsregionen) (Li et al. 2015a). At timepoint 1, the cohort included 101 welders from small- to medium-sized welding companies and 127 age-matched controls working in food-storage facilities, or as gardeners and janitors for a municipality. The control group had no exposure to welding fumes, and little or no exposure to particles (Li et al. 2015a). All individuals were non-smokers for at least six months at recruitment. A follow-up was conducted in 2016/2017 (timepoint 2). By that time, 23% of welders (N = 23) and 24% of controls (N = 31) dropped out, mainly due to the closing of one of the companies or retirement. Five current smokers were identified at follow-up (two welders, three controls), all of whom participated in the study during timepoint one and were kept part of the study cohort for consistency reasons. An additional 70 welders and 40 controls were recruited. In total, the study cohort comprises 338 individuals: 171 welders and 167 controls. We examined 142 individuals (78 welders, 96 controls) at both timepoints 1 and 2. The remaining 164 individuals (93 welders, 71 controls) were examined at a single timepoint.

During the examination, participants were asked to fill out a questionnaire, including questions about diet, alcohol intake, smoking history, use of snus (Swedish moist tobacco), physical activity, personal and family medical CVD history, and hobby exposure to particles (Li et al. 2015a).

BD vacutainers were used to collect blood samples for serum analysis. Samples were allowed to clot at room temperature for 10 min followed by centrifugation at 2400 ref for 10 min. After separation of the serum, samples were kept on dry ice for transportation to the laboratory and stored at -80 until analysis.

### Exposure assessment

A structured questionnaire was used to gather further information about occupational history (past and present workplaces), duration and type of job, and exposure to diesel or welding fumes. Detailed information about each participating welding company can be found in an earlier publication (Hedmer et al. 2014), mild steel was used almost exclusively by all welders and gas metal arc welding is the most predominant method used, which includes metal arc active gas and metal arc inert gas welding. Welders were asked about the type of welding they perform at work, the number of hours they spend welding (average per work week), their use of point-source or area-level exhaust, and their use of personal protection equipment while welding.

### Personal respirable dust measurements

The OEL for welding exposure in Sweden falls under the OEL for inorganic respirable dust since the particles in welding fumes are usually around 0.5 µm (Antonini et al. 2003; Hedmer et al. 2014). Respirable dust was sampled throughout entire welding shifts, therefore particles were also picked up from tasks other than just welding, e.g. metal grinding.

Personal sampling of respirable dust was performed for the welders using collection devices within their breathing zone, while area-level dust monitoring was used for the controls. Details can be found in earlier publications (Gliga et al. 2019; Hedmer et al. 2014; Li et al. 2015b). Following a validated method (US EPA 2018) to determine the respirable dust, the filtered samples were gravimetrically analyzed, with a detection limit of 0.05 mg/sample.

At timepoint 1, respirable dust concentration measurements were obtained for 53 of the 101 welders. For the remaining 48 welders, we estimated exposure based on welders working on similar work tasks at the same company. At timepoint 2, the respirable dust concentration was measured for 103 of the 145 welders. About half of the welders used powered air purifying respirators, for which a correction factor of 3 was used to mimic the actual exposure and not the exposure outside of the respirator. To determine the correction factor air from outside and inside the respirator was sampled, and it was concluded that the concentration of respirable particles was at least three times lower inside of the mask compared to the outside air, further information can be found in earlier publications (Hedmer et al. 2014; Li et al. 2015b). Five welders had changed their personal protective devices by timepoint 2. Four welders had a newer version of a powered air-purifying respirator including a double visor (correction factor of 50), while one welder had upgraded to a half mask (correction factor of 2).

For calculating the cumulative exposure and for the statistical analysis, the adjusted respirable dust concentrations were used to reflect the actual exposure.

### Cumulative dose

The cumulative dose for timepoint 1 was estimated by multiplying the reported years of welding experience with the respirable dust exposure (adjusted for personal protective equipment). A similar calculation was performed for timepoint 2. The estimated value from timepoint 1 was added to timepoint 2 to calculate the cumulative dose:

Cumulative dose timepoint_1 = Respirable dust timepoint_1 × Years welding timepoint_1.

Cumulative dose timepoint_2 = Cumulative dose timepoint_1 + [Respirable dust timepoint_2 × (Years welding timepoint_2 – Years welding timepoint_1)].

### Serum proteins

The Proseek Multiplex Cardiovascular II panel, a proximity extension assay from Olink Proteomics (Uppsala, Sweden) was used to measure 92 unique proteins related to cardiovascular processes in 1 μL of serum. Of the 92 proteins, three were discarded from further statistical analysis due to their low quality (BNP, ITGB1BP2, PAR1). Quality control, normalization, and processing were performed as previously described (Assarsson et al. 2014).

The diseases and biological functions of the proteins were annotated using the Ingenuity Pathway Analysis software (Ingenuity Systems, Redwood City, CA, USA). Within the top 100 annotations, nine were directly involved in CVD or related processes: disorder of blood pressure (rank #21; 41 proteins involved), early-onset hypertension (#25; 20 proteins), early-onset preeclampsia (#26; 19 proteins), late-onset preeclampsia (#28; 14 proteins), hypertension (#29; 38 proteins), chronic inflammatory disorder (#48; 40 proteins), preeclampsia (#65; 28 proteins), atherosclerosis (#75; 26 proteins), and infarction (#97; 24 proteins) (Supplementary Table 3).

### Statistical analysis of differences between the study groups

The characteristics of the study participants are presented as percentages for the categorical variables (county of birth; education; residence; exposure to particles from a hobby; smoking history; current smoking status; use of snus; alcohol, vegetable, and fish intake; physical activity; CVD history; and family CVD history) and medians and 5–95th percentiles for continuous variables (age, welding years, respirable dust, cumulative exposure, and body mass index [BMI]). The Kruskal–Wallis rank sum test was used to assess the differences in continuous variables between groups (three or more groups), followed by Dunn’s post-hoc test. For comparisons between two groups of continuous variables, the paired-samples Wilcoxon test was used. For categorical variables, Fisher’s exact test was used.

### Principal component analysis

Heatmaps for the principal component analysis were built with the prince.plot function using the *swamp* package in R v.4.3.1 (R Foundation for Statistical Computing, Vienna, Austria). First, the principal components that explain part of the variation in the protein data set were determined. This was followed by testing each variable against the components to determine possible correlations. The correlations were shown as heatmaps using the –log_10_ transformed p values. With the help of the hclust function, a hierarchical clustering of the variables was created. Based on the significant correlations shown in the heatmap, fixed factors for the linear mixed models were selected, next to CVD risk factors known from literature.

### Evaluation of differentially abundant proteins

To evaluate the associations of the cardiovascular process–related proteins and the groups (welders or controls), a longitudinal mixed model analysis was employed using the lmer function in the *lme4* package in R. The participants were included in the mixed models as random factors (random intercepts), and age, BMI, and group were used as fixed factors. The RsqGLM function from the R package *MuMin* was used to calculate the variance that is explained by fixed factors (R^2^m) and random factors (R^2^c). An adjustment for multiple comparisons was applied using the Benjamini and Hochberg false discovery rate (FDR) method (Benjamini and Hochberg 1995). A sensitivity analysis was performed for the non-smokers only (welders n = 156, controls n = 161).

Mixed models were used to evaluate the associations between measurements of exposure (separate analyses for welding years, adjusted respirable dust in mg/m^3^, and cumulative exposure in years) in welders and their cardiovascular processes–related proteins. Welders were included as random factors (random intercepts), with age, BMI, and the exposure variables as fixed factors. An adjustment for multiple comparisons was made using the Benjamini and Hochberg FDR method (Benjamini and Hochberg 1995). A sensitivity analysis was performed for the non-smokers only (n = 156).

All analyses were performed using R unless otherwise stated.

### Ethics

The study was carried out in accordance with the 1964 Helsinki Declaration.

All participants gave their oral and written consent to take part in the study. The Regional Ethical Committee of Lund, Sweden, approved the study (2010/32).

## Results

### Characteristics of the study participants

The lifestyle factors and demographics of the study participants can be found in Table 1. The welders were significantly more physically active (classified as moderate or high physical activity at least three times a week for 30 min) at timepoint 2 than at timepoint 1. Fewer welders reported a family history of CVD at timepoint 2. There were no other significant differences between timepoints, exposure groups, or between study participants and dropouts. After adjusting for personal protective equipment, the median exposure to respirable dust was 0.7 mg/m^3^ at both timepoints (5–95% timepoint 1: 0.2–4.2 mg/m^3^; timepoint 2: 0.1–2.4 mg/m^3^).

**Table 1:** Characteristics of the study participants. P-values refer to comparison between timepoints 1 and 2

	Timepoint 1		Timepoint 2		p-value welders	p-value controls
	Welders (n = 101)	Controls (n = 127)	Welders (n = 145)	Controls (n = 134)		
Continuous variables—median (5th–95th percentile)						
Age	41 (23–60)	43 (23–56)	47 (27–64.8)	48 (29–62)	–	–
Years welding	7 (1–24)	0 (0–11.7)	11 (2–30)	0 (0–9)	–	–
Respirable dust (mg/m^3^)	0.7 (0.2–4.2)	–	0.7 (0.1–2.4)	–	0.353	–
Cumulative exposure (mg/m^3^/ years)	4.6 (0.4–46.7)	–	8.4 (0.8–35.8)	–	0.060	–
Body mass index (kg/m^2^)	27.7 (21.8–34.4)	27.1 (22.4–33.9)	28.5 (22.6–37.1)	27.8 (22.2–34.7)	0.168	0.160
Categorical variables—n (%) relative to the total valid answers						
Country of birth (Sweden)	74 (73)	119 (94)	99 (69)	122 (92)	–	–
Education (university or higher)	4 (4)	17 (13)	10 (7)	18 (14)	0.197	0.532
Residence (large and small cities)	26 (26)	57 (45)	23 (16)	63 (47)	0.074	0.71
Exposure to particles from a hobby	29 (29)	20 (16)	34 (24)	25 (19)	0.377	0.518
Smoking history (ever smoked)	47 (47)	44 (35)	63 (44)	51 (38)	0.697	0.609
Smoking status (current)						
Non-smoker	96 (95)	124 (98)	135 (94)	128 (96)	0.642	0.505
Social smoker	5 (5)	3 (2)	6 (4)	3 (2)	–	–
Smoker	0 (0)	0 (0)	3 (2)	2 (2)	–	–
Current snus use	28 (28)	24 (19)	45 (31)	28 (21)	0.554	0.666
Alcohol intake (3 + times/ week)	2 (2)	4 (3)	3 (2)	5 (4)	0.79	0.919
Vegetable intake (5 + times/week)	59 (58)	82 (65)	83 (58)	95 (71)	0.733	0.685
Fish intake (3 + times/week)	2 (2)	3 (2)	13 (9)	12 (9)	0.549	0.483
Physical activity (moderate to high)	39 (39)	53 (42)	69 (48)	64 (48)	0.046	0.767
CVD history						
In the last six months	19 (19)	13 (10)	25 (17)	21 (16)	0.642	0.113
More than six months ago	23 (23)	25 (20)	45 (31)	38 (28)		
Family CVD history	44 (44)	46 (36)	35 (24)	38 (28)	0.002	0.187

Very few of the controls have had had previous exposure to welding fumes at prior occupations (T1 n = 27 ranging from 1–23 years; T2: n = 30 ranging from 1 to 23 years), however the median exposure in welding years for the control group at both points remained 0 years (Table 1).

Crude analysis without any adjustments was performed revealing a suboptimal fit of the model. To evaluate the extent to which the variation in serum levels of the 89 cardiovascular process–related proteins could be explained by the characteristics of the study participants, principal component analyses were performed separately for timepoints 1 and 2 (data not shown). The most significant parameter explaining the protein variation was age followed by BMI; thus, in addition to age and smoking history (known risk factors for CVD in the literature), BMI was included as a covariate for the statistical analysis.

### Welding and differentially abundant cardiovascular proteins

**Fig. 1:**
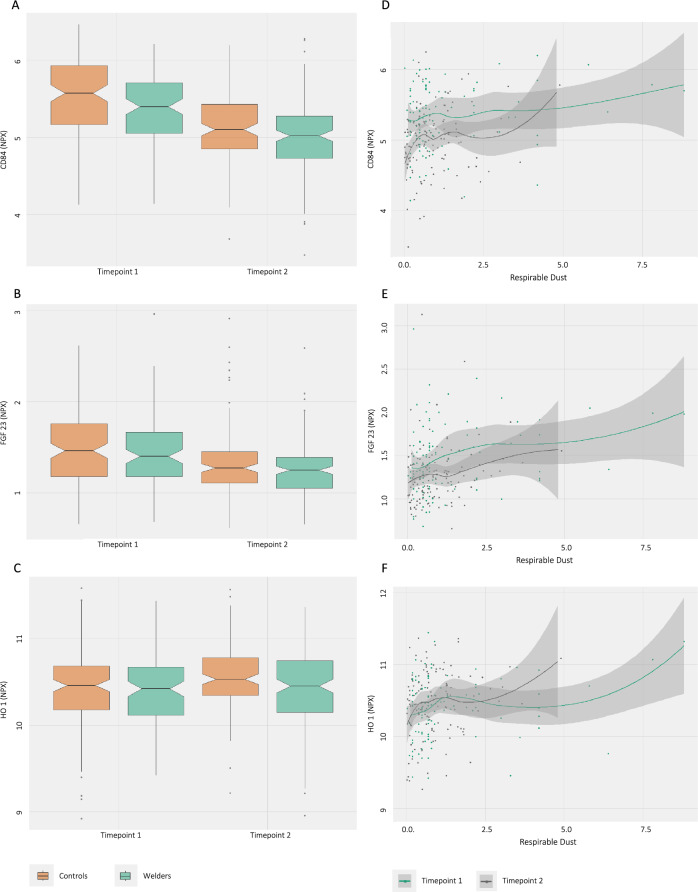
Boxplots (**A–C**) and scatterplots (**D–F**) of the serum concentrations of CD84, FGF23, and HO1. A–C show the normalized protein abundance (NPX) between welders (green) and controls (orange) separately for timepoint 1 (left side) and timepoint 2 (right side). Plots D–F show the NPX in relation to respirable dust (mg/m^3^) divided into timepoint 1 (turquois) and timepoint 2 (gray). The gray shaded area shows the predicted 95% confidence interval

Our longitudinal analysis identified ten proteins (Table 2, Supplementary Table 1) that were differentially abundant between the welding and control groups, of which one protein, cluster of differentiation 84 (CD84), remained significantly different after adjustment for multiple testing (Fig. 1). The effect estimates (β values) were all negative when comparing between exposure groups, meaning lower protein levels in welders compared with the controls.

**Table 2:** Differentially abundant proteins in serum in relation to working as a welder (left side) and being exposed to respirable dust (in welders only) (right side) and linear mixed effect models. P values are adjusted with the Benjamini–Hochberg adjustment

Protein	Welders // Controls (n = 505)				Welders // Respirable Dust (n = 222)			
	R^2^m (%)	β (SE)	p-value	adj. p-value	R^2^m (%)	β (SE)	p-value	adj. p-value
FGF23	2	− 0.06 (0.03)	0.068	0.481	7	0.08 (0.02)	< 0.001	0.002
CEACAM8	3	− 0.13 (0.06)	0.043	0.423	8	0.11 (0.04)	0.004	0.028
CD40L	2	− 0.27 (0.11)	0.019	0.340	6	0.19 (0.06)	0.004	0.028
PGF	2	− 0.05 (0.03)	0.041	0.423	8	0.05 (0.02)	0.003	0.023
CXCL1	3	− 0.11 (0.04)	0.011	0.274	7	0.06 (0.03)	0.011	0.045
CD84	4	− 0.16 (0.04)	< 0.001	0.037	10	0.09 (0.02)	0.001	0.008
HO1	1	− 0.08 (0.04)	0.043	0.423	3	0.06 (0.02)	0.013	0.050

Within the welding group, a longitudinal analysis of the different measures of exposure to welding fumes showed significant associations with protein abundance. For respirable dust, 24 of the 89 proteins (Table 2, Supplementary Table 1) showed a significant longitudinal association with the exposure after adjustment for multiple comparisons. In the sensitivity analysis, the association remained significant for 19 of those proteins (Supplementary Table 1). The effect estimates for respirable dust exposure were positive, meaning a positive association with exposure.

Seven proteins were differentially abundant in association with the respirable dust experienced by welders and also differentially abundant in welders versus the controls (Supplementary Fig. 1): fibroblast growth factor 23 (FGF23), CEA cell adhesion molecule 8 (CEACAM8), CD 40 ligand (CD40L), placental growth factor (PGF), chemokine ligand 1 (CXCL1), CD84, and heme oxygenase (HO1) (Fig. 1, Supplementary Fig. 2).

Exposure measured as welding years was associated with five differentially abundant proteins in both the main and sensitivity analyses (Supplementary Table 1). None of the changes in protein abundance remained significant after the adjustment for multiple comparisons. Changes in two proteins (serine protease 27 (PRSS27) and cathepsin L1 (CTSL1)) were associated with both the respirable dust exposure and welding years (Supplementary Fig. 1).

For cumulative exposure, 15 proteins were differentially abundant in the main analysis, and 14 in the sensitivity analysis (Supplementary Table 1). None of the changes in protein abundance remained significant after adjustment for multiple comparisons. There was an overlap of 12 proteins associated with both respirable dust exposure and cumulative exposure, and one protein (CD40L) with cumulative exposure, respirable dust exposure, and welders compared with the control group (Supplementary Fig. 1).

### Annotation of the differentially abundant proteins

The six proteins that were differentially abundant in welders and controls and also showed abundance changes in welders in association with respirable dust exposure were linked to specific CVD or related functions (Supplementary Table 3). Four proteins (CXCL1, CD84, HO1, and PGF) were linked to disorders of blood pressure, including (early-onset) hypertension. FGF23, HO1, PGF, and CD40L were related to cardiovascular damage, such as atherosclerosis and infarction. Furthermore, CD40L, CXCL1, and PGF were linked to chronic inflammatory disorders.

## Discussion

In this study, we found longitudinal differences in the protein levels of CVD-related proteins between welders and controls. We also observed changes in the same proteins over time in association with different measures of exposure to welding fumes. Importantly, we made these observations at average respirable dust exposure levels below 1 mg/m^3^, which is the lower range of current European and North American OELs, and well below the current Swedish OEL of 2.5 mg/m^3^.

### Welding and CVD-related proteins

We found that being a welder was associated with significantly lower levels of CD84 protein compared with the controls. The proteins CXCL1, PGF, FGF23, CD40L, HO1, and CEACAM8 were also present in lower levels in welders than in the controls; however, the associations for those proteins did not remain significant after adjustment for multiple comparisons. In relation to respirable dust exposure, all seven proteins showed a significant increase in protein abundance in welders. Amongst welders with previous CVD history we were able to observe a stronger association in particular with respirable dust composure compared to welders without CVD history (Supplementary Table 2). The reasons for the contrasting (negative and positive) associations in relation to the exposure to respirable dust could be a response mechanism that results in lower abundances over time, or a healthy worker effect. Similar effects could be observed within the same cohort when looking at proteins associated with cancer processes (Gliga et al. 2019) and neurological functions (Gliga et al. 2020). Six (CD84, CXCL1, PGF, FGF23, HO1, and CD40L) of these seven proteins were linked to CVD and chronic inflammatory disorders when looking at their (disease) functions (Supplementary Table 3).

To the best of our knowledge, it is not known whether any of the differentially abundant proteins identified are predictive of the development of CVD; however, they have been linked to CVD in various ways. CD84 is a member of the signaling lymphocyte activation module (SLAM) family. In various forms of CVD, it is upregulated, including in patients with acute stroke (Schuhmann et al. 2020) and in inflammatory cells in the arterial tissue of patients with Kawasaki disease (Reindel et al. 2014).

### Function of proteins linked to exposure to welding fumes

CXCL1 is primarily expressed in macrophages, neutrophiles, and epithelial cells. Elevated protein levels indicate an inflammatory reaction (Sawant et al. 2016) and are associated with CVD, such as atherosclerosis, hypertension, chronic ischemic heart disease, arterial fibrillation, and sepsis (Korbecki et al. 2022). Similar to our study, an exposure study of chimney sweeps showed a negative association (lower protein levels) in workers compared with the controls (Alhamdow et al. 2019). An earlier study focusing on asbestos exposure also showed a decrease in CXCL1 protein abundance in workers (Comar et al. 2014).

PGF has several functions, including acting as a proinflammatory cytokine, promoting plaque formation, and as a mitogen for endothelial cells (Chen et al. 2020). Higher PGF values have been associated with an increased risk of CVD (Maglione et al. 1991; Santalahti et al. 2017). PGF has also been shown to stimulate angio- and arteriogenesis and promote endothelial cell growth and survival (Kim et al. 2011; Ziche et al. 1997). The relation between lower level of PGF in welders compared with controls and toxic outcomes is thus unclear.

CD40L is involved in atherogenesis and the inflammatory response, during which it increases the release of reactive nitrogen and oxygen species (Chakrabarti et al. 2005). High CD40L levels are significantly correlated with increased levels of local inflammation in patients with atherosclerosis (Aggarwal et al. 2004), and studies have also shown elevated levels in patients with unstable coronary diseases (Aukrust et al. 1999; Garlichs et al. 2001). Changes in CD40L protein abundances were also linked to the increased air pollution during the Chinese Olympics (Altemose et al. 2017), and an earlier study showed a significant increase in its abundance in association with PM_2.5_, sulfate, elemental carbon, and sulfur dioxide exposure (Zhang et al. 2013). In type 2 diabetics, a decrease of soluble CD40L was associated with exposure to elemental carbon (Stewart et al. 2010). Increases in CD40L protein, but not mRNA abundance, have been associated with particulate matter in relation to CVD (Chen et al. 2018).

HO1 has antioxidant functions and acts as a catalyst in degrading heme into its components. It also functions as an anti-inflammatory and anti-apoptotic enzyme. HO1 prevents vascular injury and takes part in maintaining antioxidant/oxidant homeostasis (Araujo et al. 2012). It was shown to have protective functions against progressing atherosclerotic disease (Kishimoto et al. 2019). In people with pre-existing CVD, HO1 abundance has been positively associated with traffic-related pollution (Wittkopp et al. 2016). An increase in the methylation of *HO1* was observed in people occupationally exposed to arsenic (Janasik et al. 2018), and recently lower serum levels of HO1 were associated with respirable dust exposure in construction workers (Gliga et al. 2023). The lower level of HO1 in the welders compared with controls may indicate a higher risk of CVD.

FGF23 is involved in the metabolism and regulation of vitamin D and phosphate. It functions in the kidneys and is secreted by osteocytes. When the renal function declines, the concentration of FGF23 increases in a stimulating manner, which results in cardiac hypertrophy (Stöhr et al. 2018). To our knowledge, there is no data on particle exposure and FGF23 abundance. The lower level of FG23 among welders in relation to CVD risk warrants further research.

Previous studies have identified various health implications of welding, such as increases in diastolic as well as systolic blood pressure (Li et al. 2015a), changes in the methylation pattern of genes associated with CVD and lung cancer development (Dauter et al. 2021), and changes in the abundance of proteins linked to cancer processes (Gliga et al. 2019). Our results further indicate that exposure to welding fumes has an impact on the levels of protein linked to CVD at low-to-moderate exposure levels.

It should be noted that there are many shared risk factors and disease mechanisms between CVD and cancer, with inflammation and altered angiogenesis being a hallmark of both diseases (de Boer et al. 2020). Additionally, it was recently reported that the leading cause of non-cancer-related death among patients with cancer is CVD (de Boer et al. 2020), with lung cancer showing the highest proportion of patients dying of any non-cancer causes (Yang et al. 2021).

### Strength and limitations

Few proteins were significant after adjustment for multiple testing. Usually, adjustments for multiple testing are performed with the assumption that the data are independent from one another; however, we are investigating proteins in similar biological pathways, so assuming independence between proteins may lead to overadjustment. Instead, compared with the p-values, effect estimates are more applicable when looking at biological relevance. We observed rather small effect estimates, and therefore our results need to be interpreted with caution.

An important strength of our study is that the participants were non-smokers (we only had three smokers in each exposure group at re-examination) as tobacco consumption is a major risk factor for CVD. Due to the comprehensive questionnaire, we also collected detailed data on several lifestyle factors of the participants. Furthermore, we used three different measures to assess welding fume exposure. However, we were only able to measure exposure once at each timepoint, therefore our respirable dust data should be interpreted with caution.

Further, a more detailed study following welders for a longer period of time would be beneficial to be able to observe the effects of welding fume exposure more long term and study the changes within the body more closely. The changes in this study cohort are observed at low exposure, it would therefore also be beneficial to perform similar studies with a wider range of exposure to be able to get a clearer picture of the outcome on cardiovascular related health.

## Conclusion

Our study indicates that exposure to mild steel welding fumes at average respirable dust exposure levels of below 1 mg/m^3^, which is lower than most European and North American OELs (1–5 mg/m^3^) and below the Swedish OEL (2.5 mg/m^3^), was associated with changes in the abundance of several proteins involved in CVD. Whether the changes in protein abundances could serve as prospective biomarkers of occupational CVD remains unclear; rather, these markers could indicate an adaptive response to respirable dust exposure in occupational settings. This is an exploratory study, but our findings indicate the need for further research to investigate the health effects of occupational welding exposure. Moreover our findings suggest potential adverse effects on CVD at low levels of exposure that should be considered when setting OELs for respirable dust/welding fumes.

## Supplementary Information

Below is the link to the electronic supplementary material.

Supplementary file1 (DOCX 655 KB)

## Data Availability

This study is based on human sensitive data and we do not have the ethical permission to share the data.
