# Supplementary material for: Longitudinal changes in cardiovascular disease–related proteins in welders
Source: Int Arch Occup Environ Health. 2024 Jul 3;97(7):803–12. doi: 10.1007/s00420-024-02086-8 (PMC11416389; doi:10.1007/s00420-024-02086-8)
Supplement: Supplementary file 1 — Supplementary file1 (DOCX 655 KB) [file 420_2024_2086_MOESM1_ESM.docx]

Supplementary Material

Longitudinal changes in cardiovascular disease–related proteins in welders

Ulrike Maria Dauter^1^, Anda Roxana Gliga^1^, Maria Albin^1,2^, Karin Broberg^1,3^

^1^ Institute of Environmental Medicine, Karolinska Institute, Stockholm, Sweden

^2^ Centre for Occupational and Environmental Medicine, Region Stockholm, Sweden

^3^ Division of Occupational and Environmental Medicine, Department of Laboratory Medicine, Lund University, Lund, Sweden

Supplementary Table 1: Differentially abundant proteins in serum and linear effect models divided into four different exposure groups; being exposed to welding fumes in comparison with unexposed controls, and in welders only where exposure was measured in welding years, adjusted respirable dust and cumulative exposure.

| Protein | R²m (%) | β (SE) | p-value | adj. p | n | R²m (%) | β (SE) | p-value | adj. p | n |
| --- | --- | --- | --- | --- | --- | --- | --- | --- | --- | --- |
|  | Adjusted model | | |  |  | Sensitivity model | | | |  |
| **Welders // Controls** | | | | | |  | | | | |
| FGF23 | 2% | -0.06 (0.03) | 0.068 | 0.481 | 505 | 2% | -0.07 (0.04) | 0.046 | 0.463 | 483 |
| IL16 | 3% | -0.11 (0.04) | 0.005 | 0.220 |  | 3% | -0.11 (0.04) | 0.007 | 0.202 |  |
| CEACAM8 | 3% | -0.13 (0.06) | 0.043 | 0.423 |  | 2% | -0.14 (0.07) | 0.033 | 0.463 |  |
| CD40L | 2% | -0.27 (0.11) | 0.019 | 0.340 |  | 2% | -0.28 (0.12) | 0.015 | 0.316 |  |
| PGF | 2% | -0.05 (0.03) | 0.041 | 0.423 |  | 2% | -0.05 (0.03) | 0.055 | 0.463 |  |
| CXCL1 | 3% | -0.11 (0.04) | 0.011 | 0.274 |  | 3% | -0.11 (0.05) | 0.018 | 0.316 |  |
| CD84 | 4% | -0.16 (0.04) | <0.001 | 0.037 |  | 4% | -0.16 (0.05) | 0.001 | 0.068 |  |
| HO1 | 1% | -0.08 (0.04) | 0.043 | 0.423 |  | 1% | -0.08 (0.04) | 0.041 | 0.463 |  |
| LPL | 3% | -0.08 (0.04) | 0.042 | 0.423 |  | 3% | -0.08 (0.04) | 0.062 | 0.463 |  |
| AGRP | 5% | -0.10 (0.04) | 0.012 | 0.274 |  | 5% | -0.12 (0.04) | 0.004 | 0.197 |  |
| **Welders // Welding years** | | | | | |  | | | | |
| BOC | 7% | -0.01 (0.00) | 0.038 | 0.677 | 245 | 7% | -0.01 (0.00) | 0.029 | 0.793 | 231 |
| TRAILR2 | 3% | -0.01 (0.00) | 0.035 | 0.677 |  | 3% | -0.01 (0.00) | 0.038 | 0.793 |  |
| PRSS27 | 6% | -0.01 (0.00) | 0.023 | 0.677 |  | 6% | -0.01 (0.00) | 0.027 | 0.793 |  |
| PSGL1 | 4% | -0.01 (0.00) | 0.011 | 0.677 |  | 3% | -0.01 (0.00) | 0.028 | 0.793 |  |
| CTSL1 | 4% | -0.01 (0.00) | 0.026 | 0.677 |  | 4% | -0.01 (0.00) | 0.045 | 0.793 |  |
| **Welders // Respirable dust (adjusted)** | | | | | |  | | | | |
| ANG1 | 6% | 0.05 (0.02) | 0.019 | 0.067 | 222 | 6% | 0.05 (0.02) | 0.020 | 0.069 | 208 |
| SRC | 4% | 0.08 (0.03) | 0.013 | 0.050 |  | 4% | 0.08 (0.03) | 0.030 | 0.085 |  |
| IDUA | 2% | 0.04 (0.02) | 0.065 | 0.154 |  | 2% | 0.05 (0.02) | 0.032 | 0.090 |  |
| TNFRSF11A | 3% | 0.04 (0.02) | 0.043 | 0.132 |  | 3% | 0.05 (0.02) | 0.011 | 0.050 |  |
| PAR1 | 4% | 0.03 (0.02) | 0.048 | 0.138 |  | 4% | 0.03 (0.02) | 0.094 | 0.188 |  |
| PRSS27 | 9% | 0.06 (0.02) | 0.008 | 0.040 |  | 9% | 0.06 (0.02) | 0.009 | 0.050 |  |
| FGF23 | 7% | 0.08 (0.02) | <0.001 | 0.002 |  | 7% | 0.07 (0.02) | <0.001 | 0.007 |  |
| SPON2 | 9% | 0.03 (0.01) | <0.001 | 0.002 |  | 8% | 0.03 (0.02) | <0.001 | 0.007 |  |
| THBS2 | 4% | 0.03 (0.01) | 0.007 | 0.040 |  | 3% | 0.03 (0.01) | 0.012 | 0.051 |  |
| PRELP | 3% | 0.02 (0.01) | 0.029 | 0.100 |  | 3% | 0.03 (0.01) | 0.011 | 0.050 |  |
| SORT1 | 10% | 0.06 (0.01) | 0.000 | 0.001 |  | 10% | 0.06 (0.01) | <0.001 | 0.000 |  |
| CEACAM8 | 8% | 0.11 (0.04) | 0.004 | 0.028 |  | 7% | 0.11 (0.04) | 0.004 | 0.033 |  |
| DCN | 3% | 0.03 (0.01) | 0.011 | 0.045 |  | 3% | 0.03 (0.01) | 0.010 | 0.050 |  |
| HBEGF | 4% | 0.06 (0.03) | 0.079 | 0.164 |  | 8% | 0.06 (0.02) | 0.010 | 0.050 |  |
| GDF2 | 7% | 0.05 (0.03) | 0.056 | 0.138 |  | 4% | 0.07 (0.04) | 0.050 | 0.134 |  |
| DKK1 | 9% | 0.06 (0.02) | 0.010 | 0.045 |  | 6% | 0.07 (0.03) | 0.016 | 0.061 |  |
| THPO | 6% | 0.05 (0.02) | 0.004 | 0.028 |  | 5% | 0.04 (0.02) | 0.012 | 0.051 |  |
| MARCO | 5% | 0.03 (0.01) | 0.042 | 0.132 |  | 5% | 0.03 (0.01) | 0.022 | 0.071 |  |
| CTSL1 | 6% | 0.05 (0.02) | 0.006 | 0.034 |  | 6% | 0.05 (0.02) | 0.008 | 0.050 |  |
| hOSCAR | 3% | 0.03 (0.01) | 0.009 | 0.044 |  | 3% | 0.03 (0.01) | 0.013 | 0.051 |  |
| CA5A | 7% | 0.13 (0.04) | 0.001 | 0.008 |  | 6% | 0.12 (0.04) | 0.002 | 0.017 |  |
| HAOX1 | 5% | 0.19 (0.07) | 0.010 | 0.045 |  | 5% | 0.17 (0.08) | 0.026 | 0.081 |  |
| CD40L | 6% | 0.19 (0.06) | 0.004 | 0.028 |  | 6% | 0.19 (0.07) | 0.004 | 0.033 |  |
| PGF | 8% | 0.05 (0.02) | 0.003 | 0.023 |  | 7% | 0.05 (0.02) | 0.005 | 0.033 |  |
| IL17D | 6% | 0.04 (0.01) | 0.001 | 0.010 |  | 4% | 0.04 (0.01) | 0.003 | 0.032 |  |
| CXCL1 | 7% | 0.06 (0.03) | 0.011 | 0.045 |  | 6% | 0.06 (0.03) | 0.021 | 0.069 |  |
| FGF21 | 8% | 0.28 (0.08) | 0.001 | 0.008 |  | 9% | 0.30 (0.08) | <0.001 | 0.009 |  |
| CD84 | 10% | 0.09 (0.02) | 0.001 | 0.008 |  | 9% | 0.08 (0.03) | 0.001 | 0.017 |  |
| PAPPA | 6% | 0.04 (0.02) | 0.032 | 0.106 |  | 7% | 0.05 (0.02) | 0.019 | 0.067 |  |
| DECR1 | 8% | 0.11 (0.03) | <0.001 | 0.003 |  | 7% | 0.10 (0.03) | 0.001 | 0.012 |  |
| MERTK | 5% | 0.06 (0.02) | 0.001 | 0.014 |  | 5% | 0.06 (0.02) | 0.001 | 0.014 |  |
| HO1 | 3% | 0.06 (0.02) | 0.013 | 0.050 |  | 3% | 0.06 (0.02) | 0.019 | 0.067 |  |
| PTX3 | 3% | 0.04 (0.02) | 0.053 | 0.138 |  | 3% | 0.05 (0.02) | 0.028 | 0.082 |  |
| ACE2 | 10% | 0.09 (0.03) | 0.004 | 0.028 |  | 8% | 0.09 (0.03) | 0.005 | 0.033 |  |
| **Welders // Cumulative exposure** | | | | | |  | | | | |
| ANG1 | 5% | 0.00 (0.00) | 0.041 | 0.262 | 222 | 5% | 0.00 (0.00) | 0.031 | 0.232 | 208 |
| SPON2 | 6% | 0.00 (0.00) | 0.009 | 0.093 |  | 5% | 0.00 (0.00) | 0.010 | 0.117 |  |
| PRELP | 3% | 0.00 (0.00) | 0.063 | 0.289 |  | 2% | 0.00 (0.00) | 0.043 | 0.276 |  |
| SORT1 | 5% | 0.00 (0.00) | 0.002 | 0.093 |  | 5% | 0.00 (0.00) | 0.003 | 0.115 |  |
| MMP7 | 3% | 0.00 (0.00) | 0.008 | 0.093 |  | 3% | 0.00 (0.00) | 0.012 | 0.117 |  |
| DCN | 4% | 0.00 (0.00) | 0.007 | 0.093 |  | 3% | 0.00 (0.00) | 0.009 | 0.117 |  |
| CA5A | 4% | 0.01 (0.00) | 0.020 | 0.163 |  | 4% | 0.01 (0.00) | 0.024 | 0.191 |  |
| HAOX1 | 5% | 0.01 (0.00) | 0.008 | 0.093 |  | 6% | 0.01 (0.00) | 0.011 | 0.117 |  |
| CD40L | 5% | 0.01 (0.00) | 0.047 | 0.277 |  | 4% | 0.01 (0.00) | 0.074 | 0.282 |  |
| ADAM TS13 | 5% | 0.00 (0.00) | 0.040 | 0.262 |  | 4% | 0.00 (0.00) | 0.046 | 0.276 |  |
| FGF21 | 6% | 0.01 (0.01) | 0.009 | 0.093 |  | 6% | 0.01 (0.01) | 0.014 | 0.125 |  |
| PAPPA | 7% | 0.00 (0.00) | 0.013 | 0.115 |  | 8% | 0.00 (0.00) | 0.005 | 0.115 |  |
| REN | 5% | 0.00 (0.00) | 0.030 | 0.225 |  | 4% | 0.00 (0.00) | 0.055 | 0.276 |  |
| DECR1 | 7% | 0.01 (0.00) | 0.001 | 0.061 |  | 6% | 0.01 (0.00) | 0.001 | 0.096 |  |
| MERTK | 4% | 0.00 (0.00) | 0.005 | 0.093 |  | 4% | 0.00 (0.00) | 0.004 | 0.115 |  |
| ACE2 | 10% | 0.01 (0.00) | 0.006 | 0.093 |  | 8% | 0.01 (0.00) | 0.008 | 0.117 |  |
| both models are adjusted for BMI, age and smoking, sensitivity models only include none smokers; adjustment with Benjamini and Hochberg method | | | | | | | | | | |

Supplementary Table 2: Differentially abundant proteins in serum in relation to respirable dust exposure divided into welders without CVD history (left side) welders with CVD history (right side) and linear mixed effect models. P values are adjusted with the Benjamini-Hochberg adjustment.

| Protein | R²m (%) | β (SE) | p-value | adj. p | n | R²m (%) | β (SE) | p-value | adj. p | n |
| --- | --- | --- | --- | --- | --- | --- | --- | --- | --- | --- |
| **Welders w/o CVD history // Respirable Dust** | | | | | | **Welders with CVD history // Respirable Dust** | | | | |
| FGF_23 | 9% | 0.08 (0.03) | 0.003 | 0.019 | 162 | 10% | 0.07 (0.03) | 0.039 | 0.045 | 60 |
| CEACAM8 | 6% | 0.08 (0.06) | 0.171 | 0.200 |  | 18% | 0.14 (0.05) | 0.006 | 0.013 |  |
| CD40_L | 7% | 0.11 (0.10) | 0.253 | 0.253 |  | 28% | 0.24 (0.08) | 0.002 | 0.008 |  |
| PGF | 7% | 0.04 (0..02) | 0.062 | 0.144 |  | 13% | 0.05 (0.02) | 0.023 | 0.033 |  |
| CXCL1 | 7% | 0.08 (0.04) | 0.025 | 0.086 |  | 15% | 0.05 (0.04) | 0.177 | 0.177 |  |
| CD84 | 8% | 0.07 (0.04) | 0.084 | 0.145 |  | 24% | 0.10 (0.03) | 0.001 | 0.008 |  |
| HO_1 | 4% | 0.06 (0.03) | 0.103 | 0.145 |  | 14% | 0.08 (0.03) | 0.013 | 0.023 |  |

Supplementary Table 3: Full list of annotation of the differentially abundant proteins (included in the protein extension assay) based on cardiovascular diseases or functions of interest.

| Cardiovascular Disease, Organismal Injury and Abnormalities | Inflammatory Disease, Organismal Injury and Abnormalities | Cardiovascular Disease, Organismal Injury and Abnormalities, Reproductive System Disease | Cardiovascular Disease, Organismal Injury and Abnormalities | Cardiovascular Disease, Organismal Injury and Abnormalities |
| --- | --- | --- | --- | --- |
| Hypertension | Chronic inflammatory disorder | Preeclampsia | Atherosclerosis | Infarction |
| ACE2 | ADM | ACE2 | ADAMTS13 | ADAMTS13 |
| ADM | RAGE | RAGE | RAGE | ADM |
| RAGE | CA5A | CCL3 | CA5A | RAGE |
| ANGPT1 | CCL17 | CD4 | CD40L | CA5A |
| CA5A | CCL3 | CD84 | F2R | CD40L |
| CCL3 | CD4 | TF | TF | F2R |
| CD4 | CD40L | FS | IgG Fc receptor II-b | IgG Fc receptor II-b |
| CD84 | CTRC | GH | FGF23 | GLO1 |
| TF | CTSL | GLO1 | GH | HMOX1 |
| IgG Fc receptor II-b | CXCL1 | KIM1 | HO1 | HSPB1 |
| FS | TF | HBEGF | NEMO | IL18 |
| GDF2 | IgG Fc receptor II-b | HO1 | IL18 | IL-1ra |
| GH | GH | IL16 | IL6 | IL6 |
| GLO1 | KIM1 | IL6 | LEP | SCF |
| KIM1 | HBEGF | LEP | LPL | LEP |
| HBEGF | HO1 | MMP7 | MMP12 | LPL |
| HO1 | NEMO | BNP | BNP | BNP |
| IL16 | IL16 | PARP1 | LOX1 | LOX1 |
| IL18 | IL18 | PGF | PAPPA | PARP1 |
| IL1RA | IL1RL2 | PRELP | PARP1 | PTX3 |
| IL6 | IL1RA | PTX3 | PGF | REN |
| LEP | IL4RA | REN | PTX3 | SRC |
| LPL | IL6 | SRC | REN | TGM2 |
| MMP7 | LEP | TIE2 | SORT1 | TM |
| BNP | GAL9 | TGM2 | SRC |  |
| LOX1 | PARP1 | THBS2 | TM |  |
| PARP1 | PDL2 | TRAILR2 |  |  |
| PGF | PGF | TNFRSF11A |  |  |
| PRELP | PIGR |  |  |  |
| PTX3 | PRSS8 |  |  |  |
| REN | REN |  |  |  |
| SRC | SORT1 |  |  |  |
| TIE2 | SRC |  |  |  |
| TGM2 | STK4 |  |  |  |
| TM | TM |  |  |  |
| THBS2 | TNFRSF10A |  |  |  |
| TRAILR2 | TRAILR2 |  |  |  |
| TNFRSF11A | TNFRSF11A |  |  |  |
|  | TNFRSF13B |  |  |  |
|  | XCL1 |  |  |  |

Welding Years

Cumulative Exposure


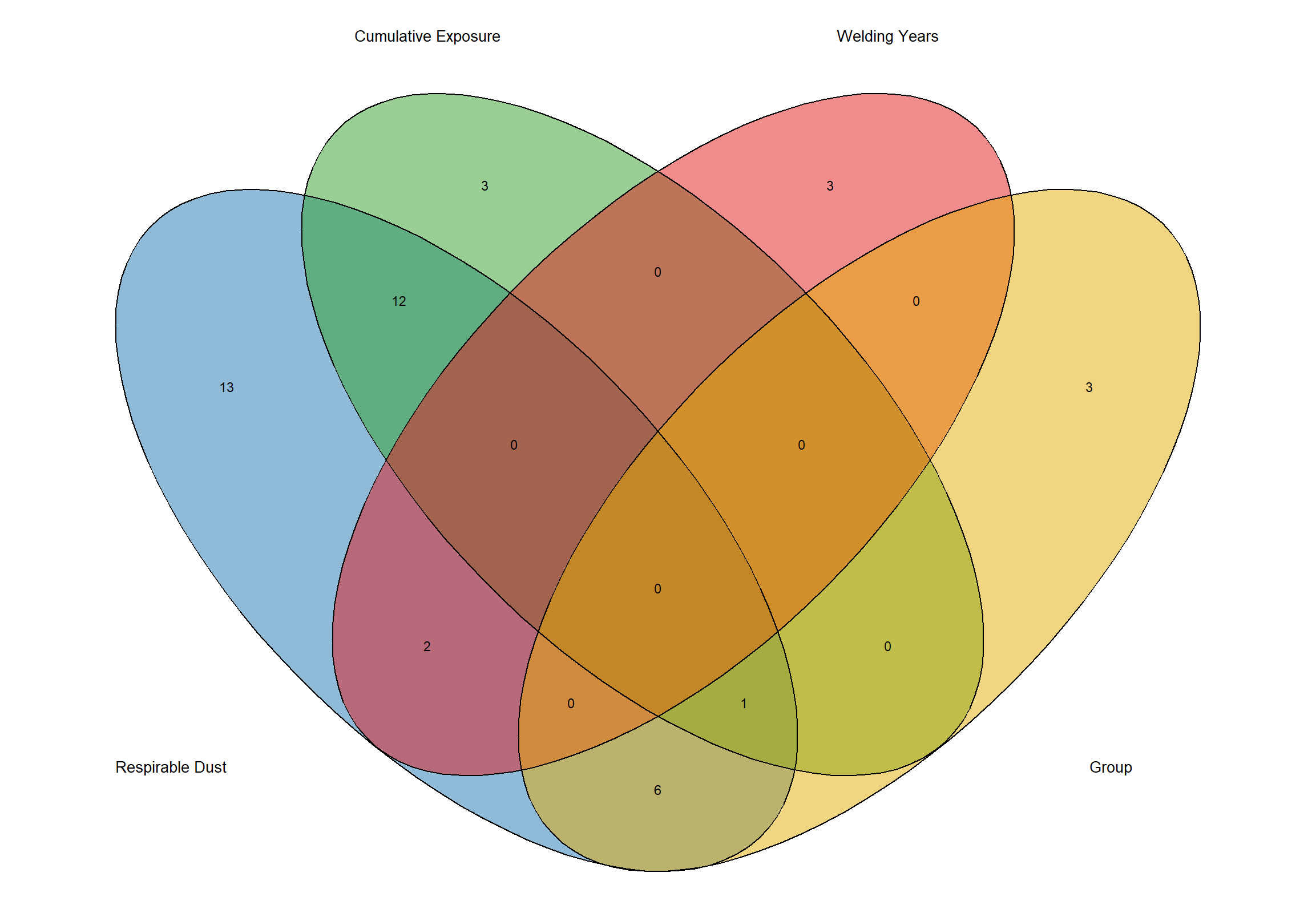


3

3

0

0

12

3

13

0

0

0

2

0

1

0

6

Exposure Group

(Controls vs Welders)

Respirable Dust

Supplementary Figure 1. Venn diagram of number of overlapping proteins for different comparisons.


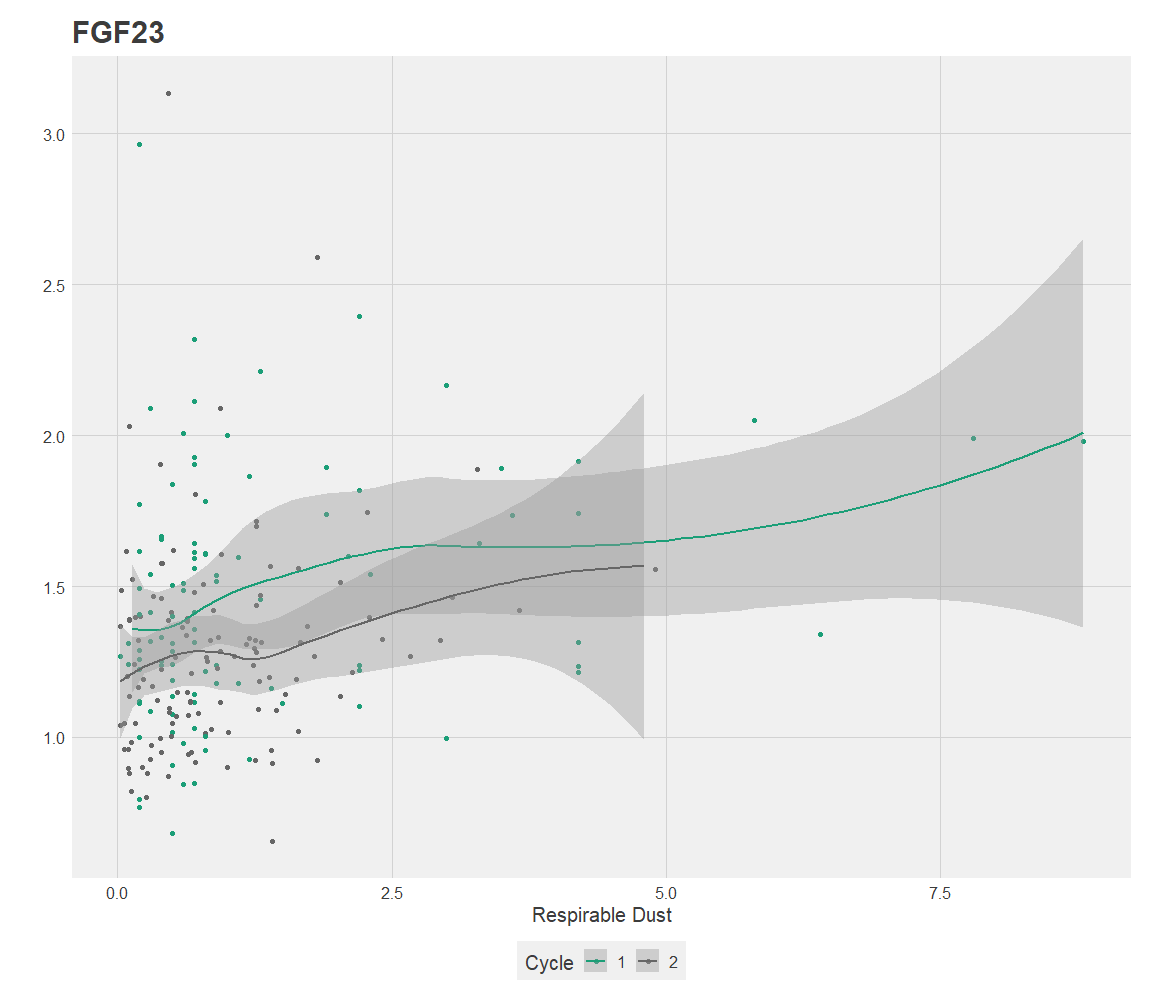

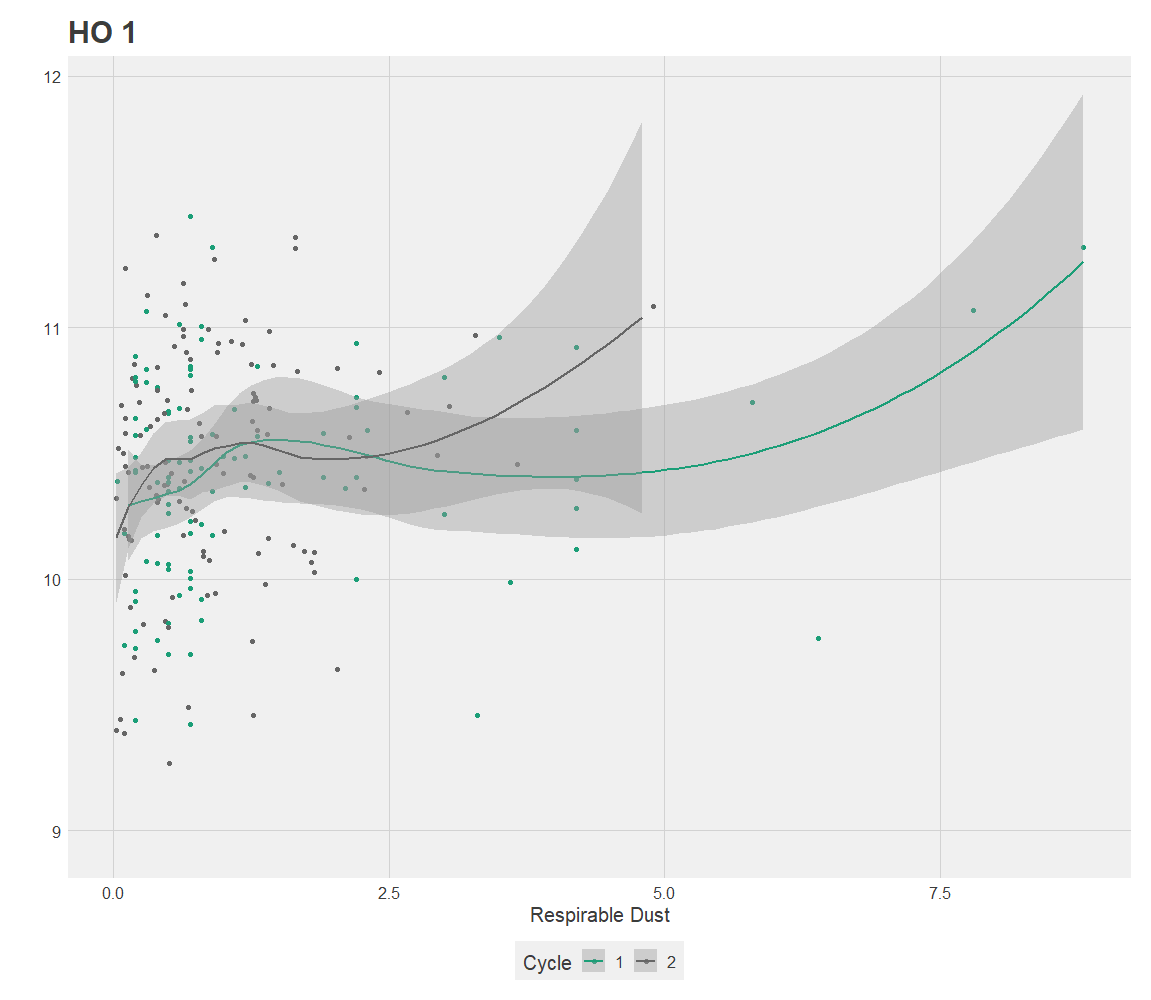

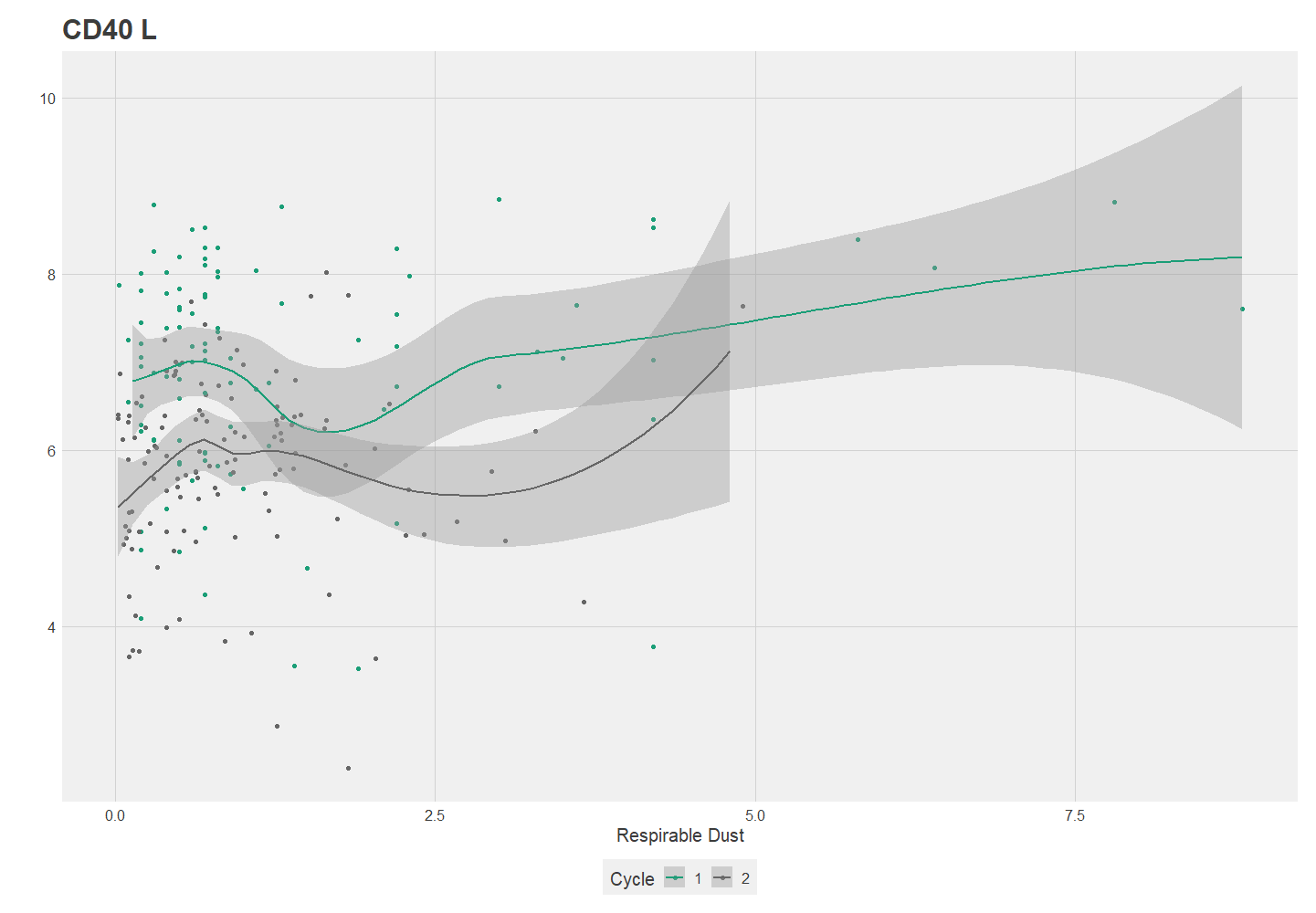

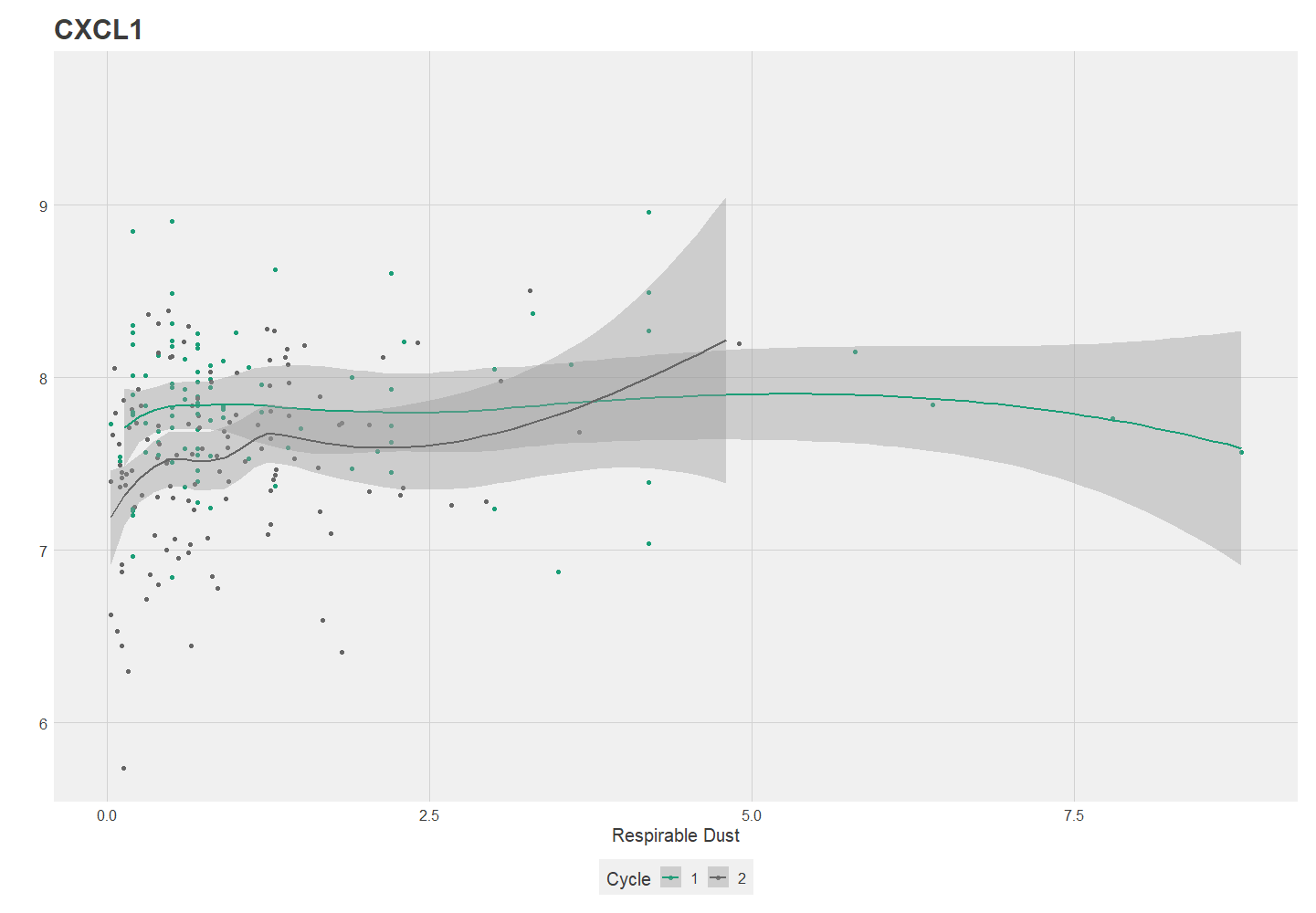


Timepoint 2

Timepoint 1

CXCL1 (NPX)

6 7 8 9

CD40 L (NPX)

4 6 8 10

Respirable Dust

0.0. 2.5 5.0 7.5

Respirable Dust

0.0. 2.5 5.0 7.5


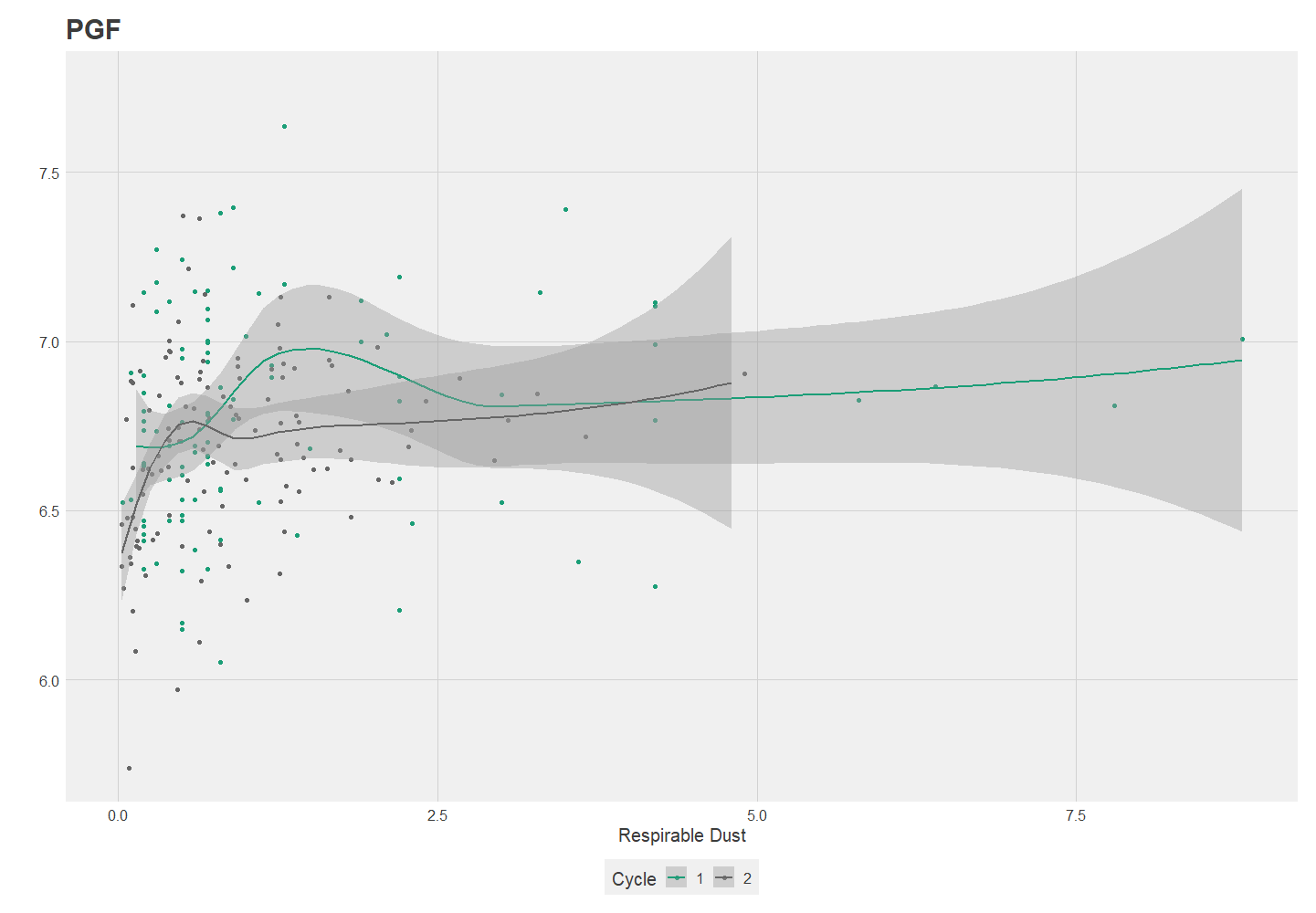

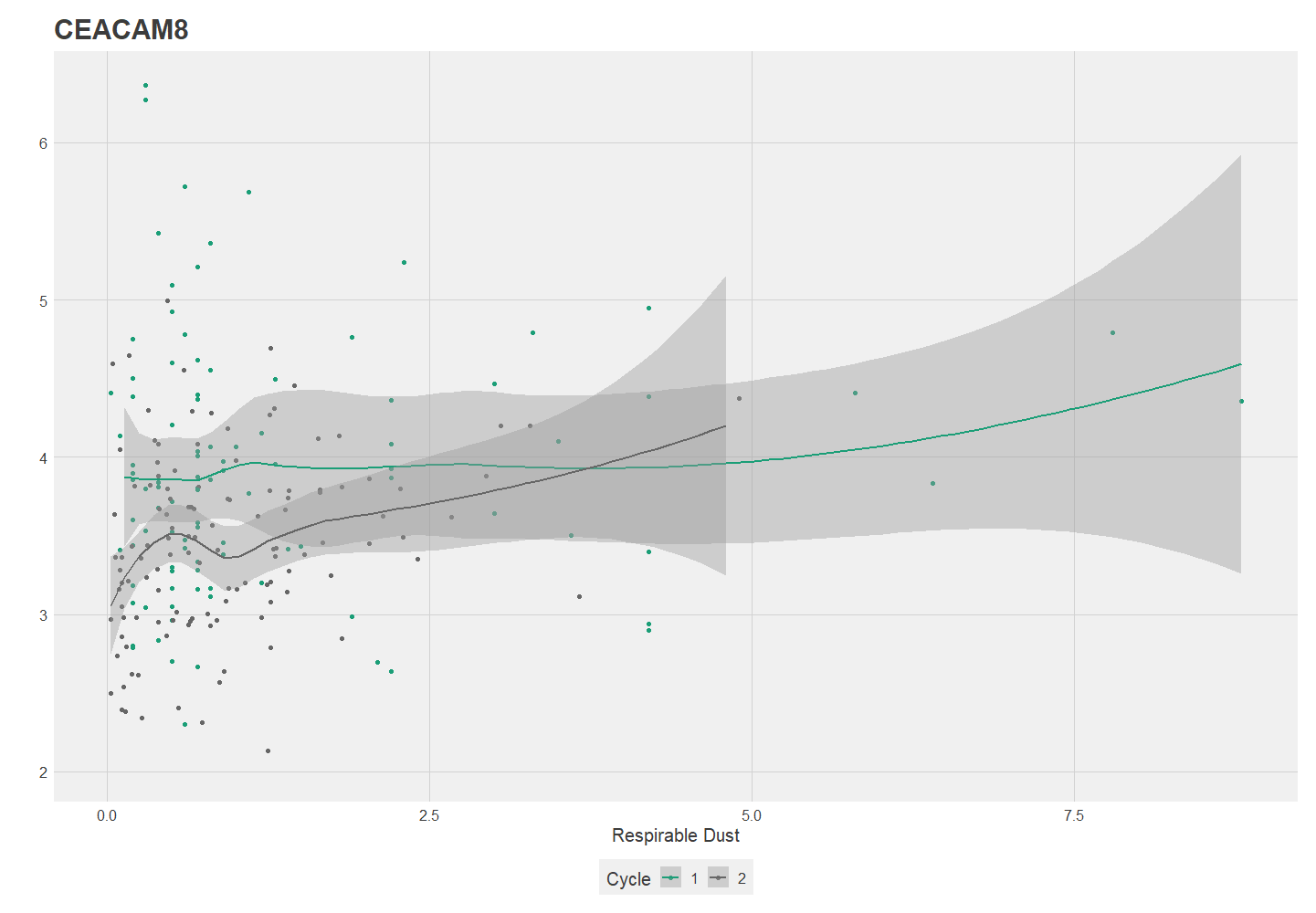


2 3 4 5 6

PGF (NPX)

6.0 6.5 7.0 7.5

Respirable Dust

0.0. 2.5 5.0 7.5

Respirable Dust

0.0. 2.5 5.0 7.5

Supplementary Figure 2: Scatterplots of serum levels of CD40L, CXCL1, PGF, and CEACAM8. Plots show normalized protein expression (NPX) in relation to respirable dust (mg/m^3^) divided into timepoint 1 (turquois) amd timepoint 2 (grey). The grey shadow shows the predicted 95% confidence interval.


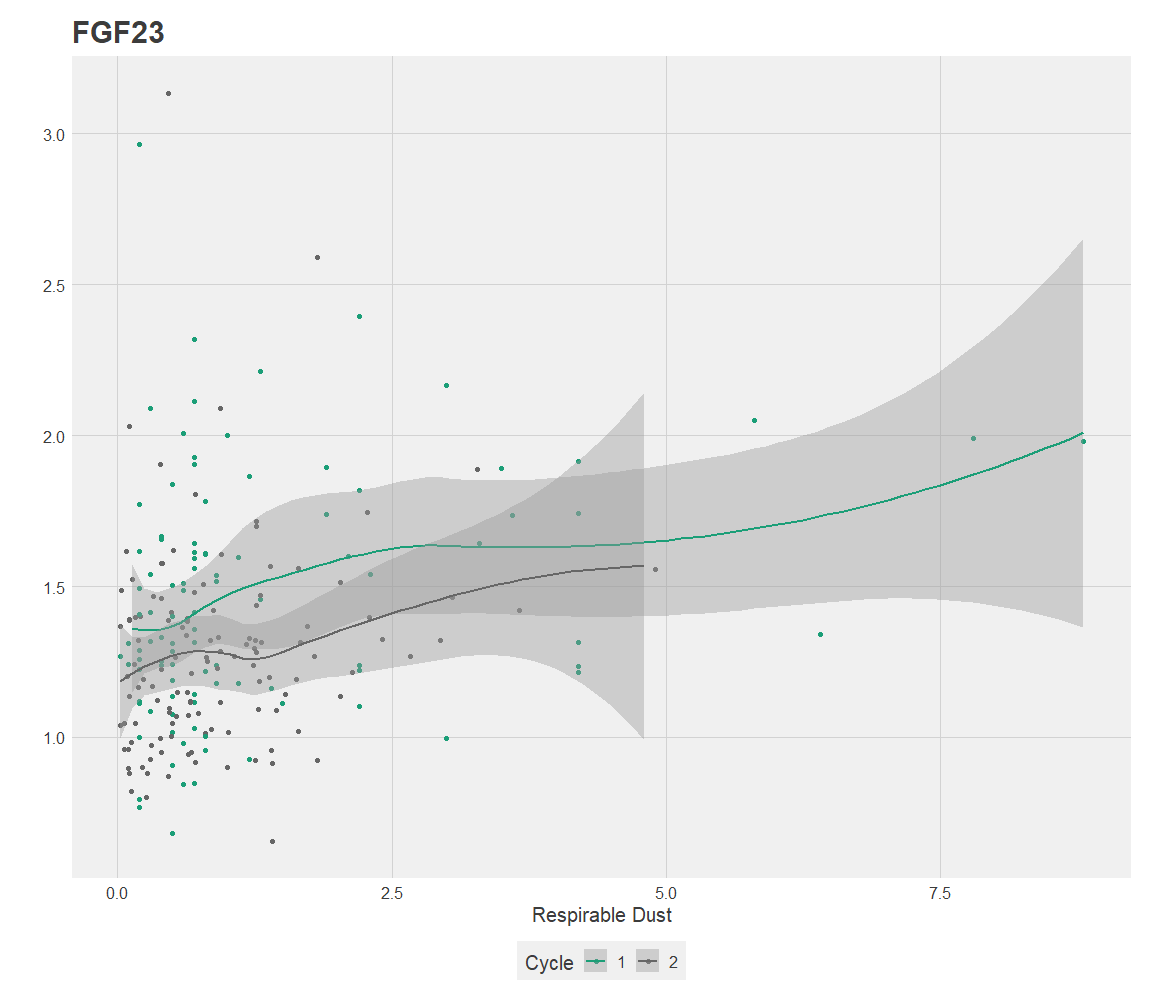

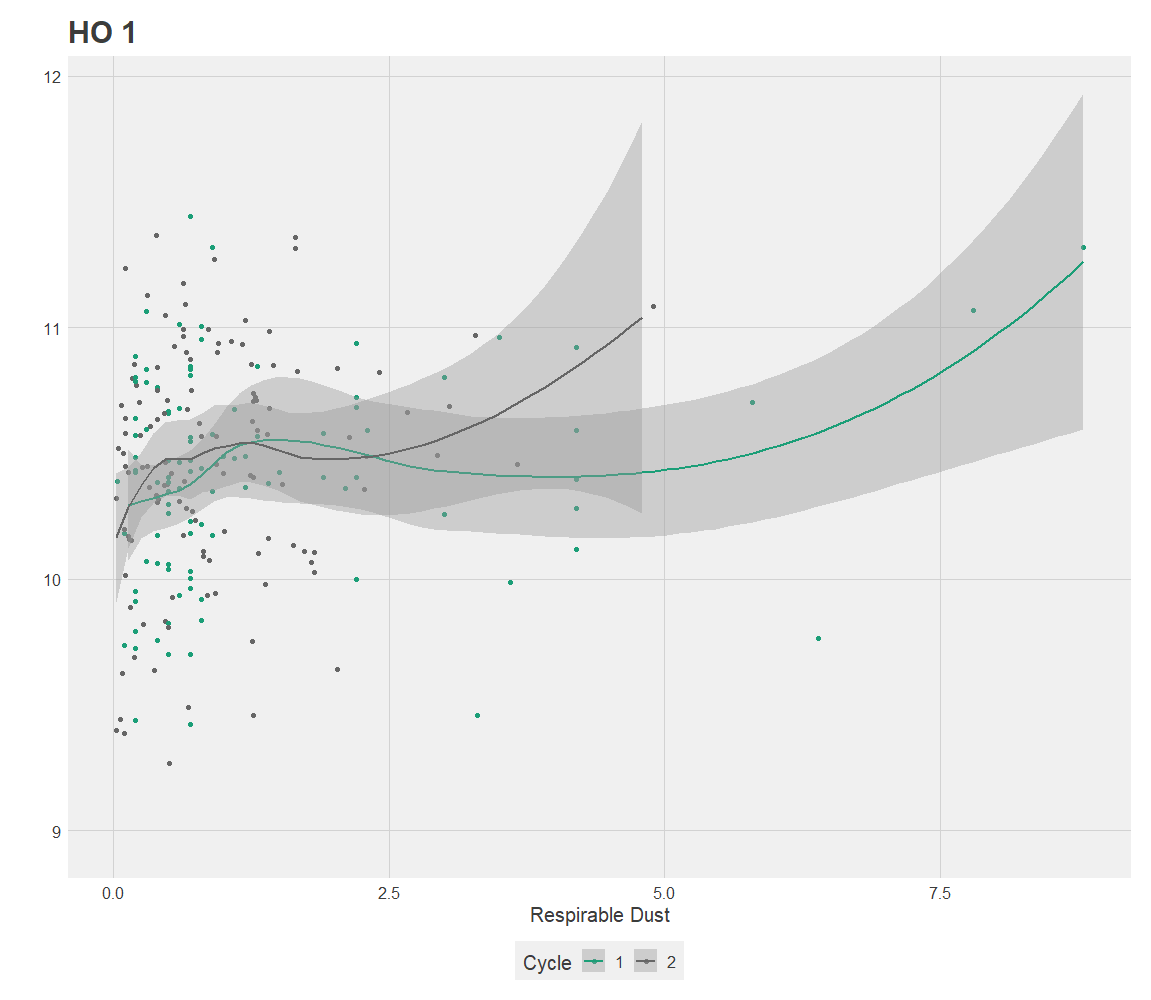


Timepoint 2

Timepoint 1

CEACAM8 (NPX)
